# The Optimal Speed for Cortical Activation of Passive Wrist Movements Performed by a Rehabilitation Robot: A Functional NIRS Study

**DOI:** 10.3389/fnhum.2017.00194

**Published:** 2017-04-20

**Authors:** Sung Jin Bae, Sung Ho Jang, Jeong Pyo Seo, Pyung Hun Chang

**Affiliations:** ^1^Department of Robotics Engineering, Graduate School, Daegu Gyeongbuk Institute of Science and Technology (DGIST)Daegu, South Korea; ^2^Department of Physical Medicine and Rehabilitation, College of Medicine, Yeungnam UniversityDaegu, South Korea

**Keywords:** functional NIRS, rehabilitation robot, cortical activation, brain plasticity, wrist rehabilitation

## Abstract

**Objectives**: To advance development of rehabilitation robots, the conditions to induce appropriate brain activation during rehabilitation performed by robots should be optimized, based on the concept of brain plasticity. In this study, we examined differences in cortical activation according to the speed of passive wrist movements performed by a rehabilitation robot.

**Methods**: Twenty three normal subjects participated in this study. Passive movements of the right wrist were performed by the wrist rehabilitation robot at three different speeds: 0.25 Hz; slow, 0.5 Hz; moderate and 0.75 Hz; fast. We used functional near-infrared spectroscopy to measure the brain activity accompanying the passive movements performed by a robot. The relative changes in oxy-hemoglobin (HbO) were measured in two regions of interest (ROI): the primary sensory-motor cortex (SM1) and premotor area (PMA).

**Results**: In the left SM1 the HbO value was significantly higher at 0.5 Hz, compared with movements performed at 0.25 Hz and 0.75 Hz (*p* < 0.05), while no significant differences were observed in the left PMA (*p* > 0.05). In the group analysis, the left SM1 was activated during passive movements at three speeds (uncorrected *p* < 0.05) and the greatest activation in the SM1 was observed at 0.5 Hz.

**Conclusions**: In conclusion, the contralateral SM1 showed the greatest activation by a moderate speed (0.5 Hz) rather than slow (0.25 Hz) and fast (0.75 Hz) speed. Our results suggest an ideal speed for execution of the wrist rehabilitation robot. Therefore, our results might provide useful data for more effective and empirically-based robot rehabilitation therapy.

## Introduction

A number of rehabilitation robots have been developed in the past two decades to aid functional recovery of impaired limbs in patients with brain injury (Volpe et al., 2000; Hesse et al., 2005; Kahn et al., 2006; Lum et al., 2006; Masiero et al., 2007; Nef et al., 2007; Coote et al., 2008; Housman et al., 2009; Chang et al., 2014). In the field of rehabilitation, high intensive, task-oriented and repetitive execution of movements is effective for functional recovery of impaired upper limbs following brain injury (Bütefisch et al., 1995; Kwakkel et al., 2004; Schaechter, 2004; Levin et al., 2008; Murphy and Corbett, 2009; Oujamaa et al., 2009). Rehabilitation robots can easily and precisely provide these labor-intensive rehabilitative treatments, and the effect of rehabilitation robots on functional recovery in patients with brain injury has been demonstrated in many studies (Volpe et al., 2000; Hesse et al., 2005; Lum et al., 2006; Masiero et al., 2007; Coote et al., 2008; Norouzi-Gheidari et al., 2012). Compared to conventional therapy (CT) provided by a therapist, the effectiveness of robot assisted therapy (RT) is questionable (Masiero et al., 2011; Norouzi-Gheidari et al., 2012). There is no difference between RT and intensive CT of the same duration/intensity condition, and extra sessions of RT in addition to CT bring better motor recovery of the shoulder and elbow (not for hand and wrist) compared with CT (Norouzi-Gheidari et al., 2012). To make the best use of robot for upper limb rehabilitation, increased efficacy of robotic rehabilitation is necessary. However, research on the optimal conditions to maximize the rehabilitative effect during treatment with a rehabilitation robot has been limited (Reinkensmeyer et al., 2007).

Brain plasticity, the ability of our brain system to reorganize its structure and function, is the basic mechanism underlying functional recovery in patients with brain injury (Schaechter, 2004; Murphy and Corbett, 2009). The underlying principle of rehabilitation in terms of brain plasticity is based on the modulation of cortical activation induced by the manipulation of external stimuli (Kaplan, 1988). Little is known about the cortical effects resulting from rehabilitation robot treatment (Li et al., 2013; Chang et al., 2014; Jang et al., 2015).

Functional neuroimaging techniques, including functional MRI (fMRI), Positron Emission Tomography (PET) and functional Near Infrared Spectroscopy (fNIRS) provide important information about the activation of the brain by external stimuli (Frahm et al., 1993; Willer et al., 1993; Miyai et al., 2001; Fujii and Nakada, 2003; Perrey, 2008; Kim et al., 2011; Leff et al., 2011; Gagnon et al., 2012). Of these, fNIRS provides a non-invasive method for measurement of the hemodynamic responses associated with activation of the cerebral cortex based on the intrinsic optical absorption of blood (Arenth et al., 2007; Irani et al., 2007; Perrey, 2008; Ye et al., 2009; Leff et al., 2011). Compared with other functional neuroimaging techniques, fNIRS has a unique advantage of less sensitivity to motion artifact and metallic material. Therefore, fNIRS appears suitable for the study of brain response during treatment with rehabilitation robots (Perrey, 2008; Mihara et al., 2010; Leff et al., 2011; Li et al., 2013; Chang et al., 2014).

In this study, we hypothesized that there exists optimal conditions for robotic rehabilitation to enhance the rehabilitative effect. The speed of movement performed by rehabilitation robot could be a unique aspect of robot rehabilitation, because varied speed can be provided consistently only with the robot. To confirm our hypothesis, using fNIRS, we examined the optimal speed of passive wrist movements performed by a rehabilitation robot that induces cortical activation through proprioceptive input by passive movements (Radovanovic et al., 2002; Francis et al., 2009; Lee et al., 2012). As a part of upper limb, the wrist enhances the usefulness of the hand by allowing it to take different orientations with respect to the elbow (van der Lee, 2001). If there exists an optimal speed that offers the greatest cortical activation, it could be applicable for robotic rehabilitation and research for other optimal conditions such as duration.

## Subjects and Methods

### Subjects

Healthy right-handed subjects (15 males, 8 females; mean age 26.5, range 21–30) with no history of neurological, psychiatric, or physical illness were recruited for this study. Handedness was evaluated using the Edinburg Handedness Inventory (Oldfield, 1971). All subjects were fully informed about the purpose of the research and provided written, informed consent prior to participation in this study. The study protocol was approved by the Institutional Review Board of the Daegu Gyeongbuk Institute of Science and Technology (DGIST). Data from two subjects were excluded because the subjects did not follow the required instructions during the data collection.

### Methods

#### Robot

**Figure 1:**
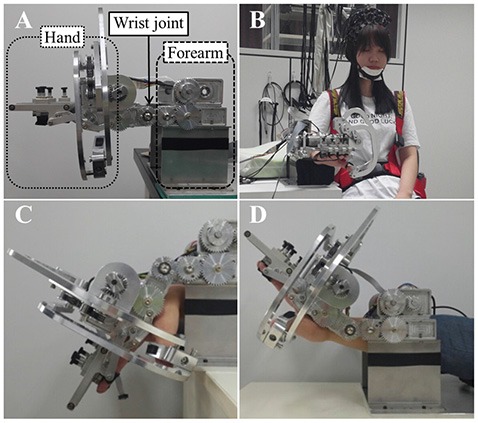
**(A)** The wrist rehabilitation robot. Lateral view of the wrist rehabilitation robot, the hand part (dotted line), wrist part (solid line) and forearm part (dashed line). **(B)** A front view of robot and subjects with the trunk strap and near infrared spectroscopy (NIRS) optodes. **(C)** Wrist flexion of the robot. **(D)** Wrist extension of the robot.

Regarding flexion and extension only, the human wrist can be simplified as a one degree of freedom (DOF) kinematic model with one revolute joint (Zatsiorsky, 2002). As mentioned above, the wrist rehabilitation robot was designed and manufactured as a simplified kinematic model of the wrist. The robot used for wrist rehabilitation has three parts: hand, wrist joint and forearm, and provides passive movement of flexion and extension (Figure 1). It has a gear driven mechanism using a single motor. The actuation system for the wrist part is composed of DC, a brushless motor with encoder (EC-i 40, Maxon motor), harmonic drive (CSF-11-50, Sam-ik THK, gear ratio 50:1), and force-torque sensor (Mini 45, ATI). In house developed software was used to control the robot. For the real-time control, Linux Fedora 11 and the Real Time Application Interface for Linux (RTAI) Ver 3.8 systems were mounted. Real-time sensing control was achieved using an encoder and Sensoray s626 board, in which time delay control (TDC) was used for precise position control. The robot showed a position error of 0.1°–1° during the experiment.

When using the robot for wrist rehabilitation, the hand and forearm must be fixed to the robot in order to perform the passive wrist movement. First, the subjects placed their forearm on the armrest made of foam covered with a soft cloth. They were instructed to place their hand on the support bar under the hand part of the robot before fixing all fingers to the finger holder with velcro straps. The robot performs the passive wrist exercise using a rotary motion of a gear driven by a motor and realizes a full range of motion (ROM) from 80° (flexion) to 75° (extension) when the degree of neutral wrist position is 0°, with the wrist in a flat position, with velocity of the wrist motions up to 2 Hz.

#### fNIRS

**Figure 2:**
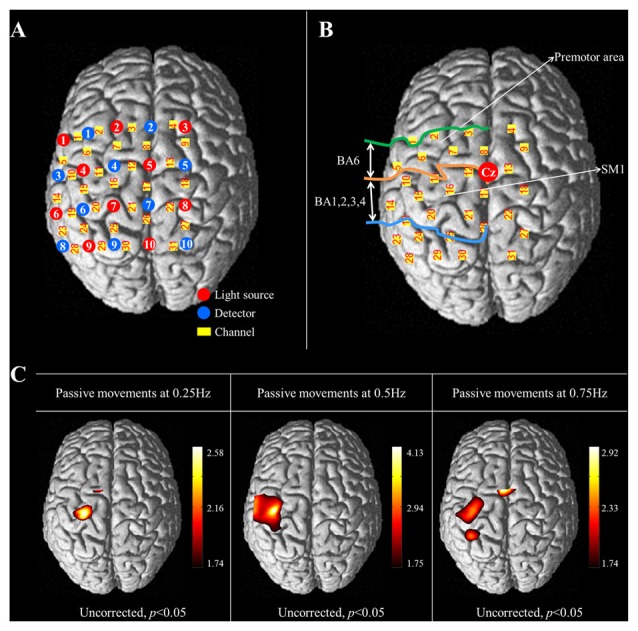
**(A)** The arrangement of NIRS optodes and channels. Twenty NIRS optodes (10 light sources and 10 detectors) are arranged in a four by five rectangular arrangement for employment of a 30 channel system. **(B)** Two regions of interest (ROI) based on Brodmann’s area (BA) and anatomical location of areas of the brain. The primary sensorimotor cortex (SM1): BA 1, 2, 3 and 4; The premotor area (PMA): BA 6. **(C)** Group-average activation map of oxy-hemoglobin (HbO) during performance of passive wrist movements by the wrist rehabilitation robot at three different speeds using NIRS-SPM (uncorrected, *p* < 0.05).

Cortical activity was measured using the fNIRS system, which has less sensitivity to motion artifacts and metallic materials than other functional neuroimaging techniques (Arenth et al., 2007; Irani et al., 2007; Perrey, 2008; Mihara et al., 2010; Leff et al., 2011). The fNIRS system (FOIRE-3000; Shimadzu, Kyoto, Japan), with continuous wave laser diodes with wavelengths of 780, 805, 830 nm, recorded cortical activity at a sampling rate of 16 Hz (Scholkmann et al., 2014). In this study, 20 NIRS optodes (10 light sources and 10 detectors) were arranged in a four by five rectangular arrangement for employment of a 30 channel system (Figure 2A). Based on the modified Beer-Lambert law, the relative changes in concentration of oxy-hemoglobin (HbO) were obtained from the optical density changes (Cope and Delpy, 1988).

The international 10/20 system was used to position the NIRS optodes. Cranial vertex (Cz) was the reference point for optode holder placement and the lines connecting T3-T4 and Nasion-Inion were used as reference lines for second row and fourth column of optodes each. The NIRS optodes was mounted on optode holder for fixation and the distance between pairs of light source-detector optodes was set at 3 cm. Using a Fastrak System (TX-2; Polhemus, Colchester, VT, USA), the coordinates of all probe positions and the anatomical landmark positions (nasion, CZ, left and right pre-auricular points) of each subject were obtained after data collection. The coordinates of the anatomical landmark and optode positions were used to estimate the position of each channel in the Montreal Neurological Institute (MNI) standard brain space (Ye et al., 2009). To examine cortical activity, two regions of interest (ROI) were selected based on Brodmann’s area (BA) and anatomical locations of brain areas; the primary sensory-motor cortex (SM1; BA1, 2, 3 and 4) and the premotor area (PMA; BA6; Figure 2B; Dassonville et al., 1998; Mayka et al., 2006; Martin, 2012). The channels included in the anatomical location of each ROI were chosen based on BA. The changes in concentration of HbO were estimated during the task phases of passive movement of right-wrist from each channel of two ROIs.

#### Procedure

Subjects were instructed to sit comfortably on a chair in upright position facing a wall in a shielded room with normal lighting during the experiment. Their trunks were firmly fixed to the chair by a strap to prevent trunk movement. They were instructed to wear the wrist rehabilitation robot on their right hand. The hand and forearm were fixed to the wrist rehabilitation robot by an operator, and the NIRS optodes were arranged on the NIRS optode holder. They were instructed to relax and not to move their wrist voluntarily before the data collection and during the performance of passive wrist movements by the wrist rehabilitation robot. While an experiment was underway, one of the operators watched the subjects to observe whether they showed active wrist movement or movement causing artifacts. We confirmed that no study subjects moved their wrists voluntarily by asking subjects after measurement. The subjects were asked to minimize physical movement of the body, including facial movement and frequent eye blinking and not to make any noise during the data recording. A block paradigm design (three cycles; with each cycle consisting of rest (20 s)—task (20 s)—rest (20 s)) was used for performance of flexion and extension movements of the right wrist by the wrist rehabilitation robot.

Three different speeds of passive wrist movements performed by the wrist rehabilitation robot (0.25, 0.5 and 0.75 Hz) were examined and the performance sequence was assigned randomly by applying a random permutation function “randperm” in MATLABR2012b (The Mathworks, Natick, MA, USA). During the experiment, the fNIRS system measured the cortical activity, while the wrist rehabilitation robot provided passive movement of right-wrist only during the task section in the block paradigm design.

#### Data Analysis

Data analysis was performed using the NIRS-SPM (Near Infrared Spectroscopy-Statistical Parametric Mapping^1^), MATLAB-based software package for statistical analysis of NIRS signals. Gaussian smoothing with a full width at half maximum (FWHM) of 2 s was applied to correct noise from the fNIRS system (Ye et al., 2009; Tak et al., 2011). The wavelet-MDL based detrending algorithm was used to correct signal distortion due to breathing or movement of the subject. General linear model (GLM) analysis with a canonical hemodynamic response curve was performed to model the hypothesized HbO response under the experimental condition (Jang et al., 2009; Ye et al., 2009; Tak et al., 2011). Hbo, which represents the amplitudes of the hemodynamic response, was tested to identify the channels that were significantly activated during the task period compared to the rest period, with a one-tailed *t* test (Plichta et al., 2006). Interpolated activity map over the cortical surface from the T statistics calculated at discrete channels were computed for group analysis, and, for a stricter analysis, HbO was considered significant at an uncorrected threshold of *p* < 0.05.

To test the role of chance in differences for the HbO value in each ROI dependent on the speed of passive wrist movements, one-way ANOVA with a least significant difference *post hoc* test was performed. The HbO values for each channel were averaged across the whole periods of task and the sums of averaged HbO values from the channels covering each ROI (SM1, PMA) was calculated in units of millimolar-millimeter. SPSS software (SPSS Inc. Released 2006. SPSS for Windows, Version 15.0. SPSS Inc., Chicago, IL, USA) was used for statistical analyses. Null hypotheses of no difference were rejected if *p*-values were less than 0.05.

## Results

**Figure 3:**
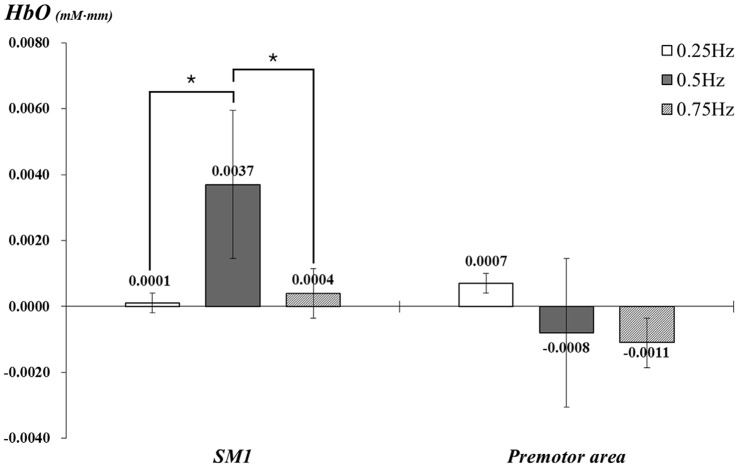
**Comparisons of HbO values in each ROI by three different speeds of passive wrist movements with standard error bar.** HbO, oxy-hemoglobin; SM1, the primary sensory-motor cortex. *p < 0.05.

With exclusion of two subjects, data for 23 subjects (mean age 26.5, range 21–30) were analyzed. In the individual GLM analysis, during passive movement of the right wrist by the wrist rehabilitation robot, in the left SM1 and PMA, HbO values changed as follows: the left SM1 −0.25 Hz: 0.0001, 0.5 Hz: 0.0037, 0.75 Hz: 0.0004 and the left PMA −0.25 Hz: 0.0007, 0.5 Hz: −0.0008, 0.75 Hz: −0.0011. In the left SM1 the HbO value was significantly higher at 0.5 Hz, compared with the value due to wrist movements performed at 0.25 Hz and 0.75 Hz (*p* < 0.05); however, no significant difference was observed between 0.25 Hz and 0.75 Hz (*p* > 0.05). In the left PMA, no difference was observed among three Hzs (*p* > 0.05; Figure 3).

In the results of group analysis, the left SM1 was activated during the passive movements performed at three different speeds (uncorrected *p* < 0.05). The greatest activation in the SM1 was observed at 0.5 Hz rather than 0.25 and 0.75 Hz (Figure 2B). In the left PMA, significant activation was observed during movement at 0.25 Hz and 0.75 Hz (uncorrected *p* < 0.05), and greater activation in the PMA was observed at 0.75 Hz than 0.25 Hz (Figure 2C).

## Discussion

In this study, we examined the differences in cortical activation at three different speeds of passive wrist movements performed by the wrist rehabilitation robot. An experiment including 1.0 Hz was performed during the pilot study; however, it was too fast to perform full ROM from 80° (flexion) to 75° (extension) of the wrist joint and several subjects could not accommodate the movements at 1 Hz speed. A previous study also reported that 1 Hz was too fast for performance of precise movements of the wrist joint in stroke patients (Szameitat et al., 2012). Therefore, the speed of movements of the wrist rehabilitation robot was categorized and defined as follows: 0.25 Hz, slow; 0.5 Hz, moderate; and 0.75 Hz, fast. To determine the ideal speed, we measured HbO, the most commonly used parameter of fNIRS (Hoshi et al., 2001; Miyai et al., 2001; Strangman et al., 2002; Wolf et al., 2002). As an index of neural activation, HbO detects the hemodynamic changes of the underlying cerebral cortex indirectly (oxygen consumption by neuronal cells; Irani et al., 2007; Perrey, 2008). The results were: (1) passive wrist movements performed by our wrist rehabilitation robot caused activation of the contralateral SM1 and PMA; mainly observed in the contralateral SM1; and (2) the greatest activation of the contralateral SM1 was observed during passive wrist movements at a speed of 0.5 Hz compared with 0.25 and 0.75 Hz.

Our wrist rehabilitation robot appeared to induce cortical activation mainly through proprioceptive input (Radovanovic et al., 2002; Francis et al., 2009; Mtui et al., 2011; Lee et al., 2012). Different amounts of proprioceptive input are evoked at three different speeds of passive wrist movements, inducing differences in cortical activation of the contralateral SM1 and PMA. Significant contralateral SM1 activation was observed in passive robot movements performed at moderate speed (0.5 Hz) rather than slow (0.25 Hz) and fast (0.75 Hz). Therefore, we suggest that sufficient proprioceptive input to the contralateral SM1 can be achieved by movement at a moderate speed.

It is noteworthy that the activation areas of our results are similar to prior research regardless of using robots: whether movements were performed with robots (Li et al., 2013; Jang et al., 2015) for normal people or without robots (Lotze et al., 2003; Szameitat et al., 2012) for both of normal people and stroke patients, the same areas were activated. Szameitat et al. (2012) reported that similar to our results, SM1 areas were activated with passive wrist movements without using a robot. More specifically, in 2012 they examined cortical activation patterns by fMRI during passive (and active) wrist movement at a speed of 1.0 Hz in 21 normal subjects and five stroke patients. Clearly our results confirm previous research work. The research employing robots likewise report that the SM1 area was activated (Li et al., 2013; Jang et al., 2015). In a recent study to determine the optimal speed for the passive hand movement by the rehabilitation robot, Chang et al. (2014) examined the cortical activations induced by hand movements performed at 0.25 Hz, 0.5 Hz and 1 Hz by a rehabilitation robot in nine healthy subjects. Significant activation was observed in the contralateral SM1 during movements performed at 0.5 Hz (Jang et al., 2015).

Earlier robot research focused mainly on the effects of RT vs. CT under the same conditions such as duration/intensity, or examined extra sessions of RT in addition to CT (Masiero et al., 2011; Norouzi-Gheidari et al., 2012). Differently from earlier robot research, however, this study focused on the optimal speed condition, which could be unique aspect of robot research, by providing various speeds consistently. To the best of our knowledge, this is the first study to report the optimal speed for cortical activation during passive movements of the wrist by a rehabilitation robot. We partially confirmed our hypothesis that there exists an optimal speed for robot rehabilitation in normal people, and the results of our study present the possibility of existence of optimal speed for stroke patients. Furthermore, our results could suggest an evaluation standard for checking the recovery of stroke patients by confirming the greatest activation with the passive movement by robot with the speed at 0.5 Hz. The limitations of this study should be considered. First, cortical activation could not be measured at more than three speeds: finer division between 0.25, 0.5 and 0.75 Hz is desired. Second, clear evidence of preventing voluntary wrist movements, such as electromyogram, could not be presented. Third, a small number of subjects participated in the experiments. More subjects should be enrolled in larger trials before the clinical application of our results for patients with brain injury such as stroke.

An immediate extension of our study would be to examine the cortical activation due to active movement with robots and to compare the results with passive movements. To elaborate, it has been reported that not only the activation areas due to passive movement and active movement are similar (Szameitat et al., 2012), but also the active movement yields significantly higher activation than the passive (Lotze et al., 2003). These two results, however, were obtained without using robots and there has been no comparable research reported using robots. In this regard, the study by Li et al. (2013) is informative in that it incorporates the active movement of elbow by a robot and observation of the cortical activation by fNIRS.

In conclusion, in examination of the optimal speed of passive wrist movements performed by a wrist rehabilitation robot at three different speeds, we found that the greatest activation of the contralateral SM1 was observed at a moderate speed (0.5 Hz) rather than slow (0.25 Hz) and fast (0.75 Hz) speed. Our results suggest an optimal condition for execution of the wrist rehabilitation robot in terms of speed. Therefore, we believe that our results might provide useful data for more effective and empirically-based robot rehabilitation therapy. In addition, fNIRS appears to be a useful evaluation tool for research on the rehabilitation robot.

## Author Contributions

SJB and SHJ contributed equally to this work and should be considered as co-first authors. SJB and JPS contributed to acquisition and analysis of data. SHJ and PHC contributed to conception and design and interpretation of data. PHC participated in drafting the article and gave the final approval of the version to be submitted.

## Funding

This work was supported by the DGIST R&D Program of the Ministry of Science, ICT and Future Planning (16-BD-0401).

## Conflict of Interest Statement

The authors declare that the research was conducted in the absence of any commercial or financial relationships that could be construed as a potential conflict of interest.

## Footnotes

^1^http://bisp.kaist.ac.kr/NIRS-SPM
